# Binomial Mitotic Segregation of *MYCN*-Carrying Double Minutes in Neuroblastoma Illustrates the Role of Randomness in Oncogene Amplification

**DOI:** 10.1371/journal.pone.0003099

**Published:** 2008-08-29

**Authors:** Gisela Lundberg, Anders H. Rosengren, Ulf Håkanson, Henrik Stewénius, Yuesheng Jin, Ylva Stewénius, Sven Påhlman, David Gisselsson

**Affiliations:** 1 Department of Clinical Genetics, Lund University Hospital, Lund, Sweden; 2 Department of Clinical Sciences Malmö, Lund University, Lund, Sweden; 3 The Nanometer Structure Consortium, Division of Solid State Physics, Lund University, Lund, Sweden; 4 Department of Mathematics, Lund University, Lund, Sweden; 5 Department of Molecular Medicine, Malmö, Lund University, Lund, Sweden; 6 Department of Pathology, Lund University Hospital, Lund, Sweden; Duke University, United States of America

## Abstract

**Background:**

Amplification of the oncogene *MYCN* in double minutes (DMs) is a common finding in neuroblastoma (NB). Because DMs lack centromeric sequences it has been unclear how NB cells retain and amplify extrachromosomal *MYCN* copies during tumour development.

**Principal Findings:**

We show that *MYCN*-carrying DMs in NB cells translocate from the nuclear interior to the periphery of the condensing chromatin at transition from interphase to prophase and are preferentially located adjacent to the telomere repeat sequences of the chromosomes throughout cell division. However, DM segregation was not affected by disruption of the telosome nucleoprotein complex and DMs readily migrated from human to murine chromatin in human/mouse cell hybrids, indicating that they do not bind to specific positional elements in human chromosomes. Scoring DM copy-numbers in ana/telophase cells revealed that DM segregation could be closely approximated by a binomial random distribution. Colony-forming assay demonstrated a strong growth-advantage for NB cells with high DM (*MYCN*) copy-numbers, compared to NB cells with lower copy-numbers. In fact, the overall distribution of DMs in growing NB cell populations could be readily reproduced by a mathematical model assuming binomial segregation at cell division combined with a proliferative advantage for cells with high DM copy-numbers.

**Conclusion:**

Binomial segregation at cell division explains the high degree of *MYCN* copy-number variability in NB. Our findings also provide a proof-of-principle for oncogene amplification through creation of genetic diversity by random events followed by Darwinian selection.

## Introduction

Neuroblastomas (NB) are tumours of the sympathetic nervous system, occurring predominantly in early childhood and accounting for 8–10% of all paediatric cancers. One of the most important prognostic markers in NB is amplification of the oncogene *MYCN*, which is observed in ≈30% of NB [1]. Amplification of *MYCN* is associated with advanced stages of disease and the 3-year event-free survival of tumours with *MYCN* amplification is <20% [2]. The MYCN protein forms a heterodimer with MAX. This protein complex functions as a transcriptional activator, the targets of which include *ODC, MCM7,* and *MDR1* [3], [4]. The activation of these genes leads to progression through the G1 phase of the cell cycle. Conversely, transcriptional repression of *MYCN,* or *MYCN* copy-number elimination, leads to growth-arrest, senescence and apoptosis of NB cells [5]–[7]. Cytogenetically, *MYCN* amplicons are typically carried either in extrachromosomal double minute (DMs) or in intrachromosomal homogeneously staining regions (HSRs). Under *in vitro* conditions HSR is the most common manifestation of *MYCN* amplification, whereas in fresh NB biopsies the amplicons are typically carried by DMs [8]. DMs are composed of circular DNA, up to only a few million base pairs in size, containing no centromere and no telomere [9]. DMs typically replicate only once during S-phase [10]. Because DMs lack centromeric sequences it has remained unclear how they segregate through mitosis and, consequently, how they are maintained and amplified in a growing NB cell population. However, recent elegant studies of the colorectal cancer model COLO322 have shown that DMs can move through mitosis by binding close to the termini of human chromosomes [11], [12]. The aims of the present study were (1) to characterise the cell cycle dynamics of DMs in NB cells and assess to which extent their behaviour is similar to that inferred by studies in other model systems, (2) to deduce the statistical principles of DM segregation, and (3) to investigate the topography of DM binding sites on human chromosomes. Based on our findings, we finally sought to assess whether unequal segregation of DMs to daughter cells at mitosis could play a mechanistic role in *MYCN* gene amplification by generating cell populations with heterogeneous DM copy-numbers on which Darwinian principles could be imposed.

## Results

### Cell cycle dynamics of DMs

**Figure 1:**
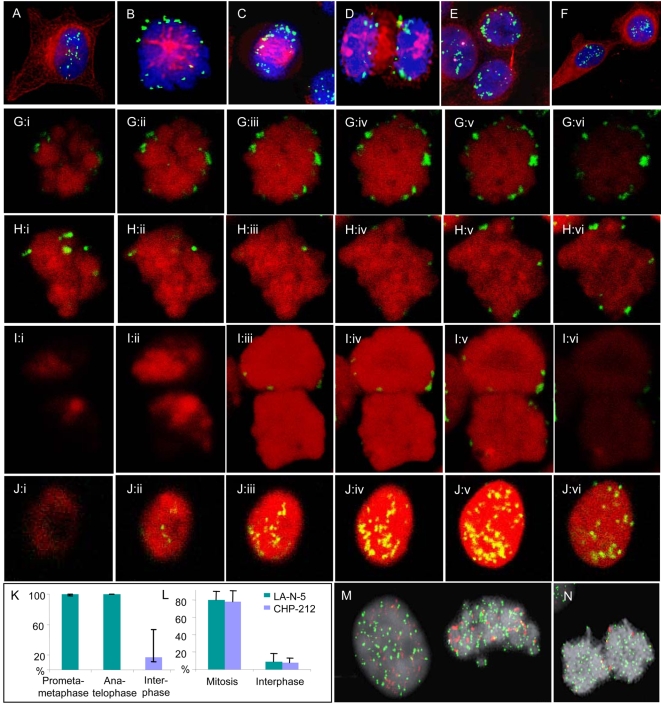
Fluorescence microscopy. All figures are from CHP-212. A–F. Combined immunofluorescence (IF) for beta tubulin (red), fluorescence in situ hybridisation (FISH) with a *MYCN* probe (green), and chromatin counterstaining by DAPI (blue) shows translocation of DMs to the periphery of the chromosome rosette at transition from interphase (A) to prometaphase (B); the peripheral localisation is maintained through metaphase (C), anaphase (D) and telophase (E), after which DMs again transfer to interior positions in the nucleus (F). G–J. Serial confocal optical sections (i–vi) corroborate the peripheral positions of DMs (green) at prometaphase (G), metaphase (H), and telophase (I), in contrast to a more central localisation at interphase (J); chromatin is counterstained by DRAQ5 (red). K. Proportion of peripheral DMs at prometa/metaphase, ana/telophase, and interphase; error bars denote standard deviation. L. The proportion of DM (*MYCN*) signals overlapping with telomeric TTAGGG-repeat signals at mitosis and interphase, respectively, in LA-N-5 and CHP-212. M–N. FISH for telomeric TTAGGG sequences (green) and *MYCN* (red) shows more frequent overlapping of signals for telomeres and DMs at metaphase (M, right) and telophase (N), compared to interphase (M, left).

To locate DMs in relation to chromosomes through the cell cycle, *MYCN* amplicons in the two NB cell lines CHP-212 and SK-N-AS were detected by combined fluorescence in situ hybridization (FISH) and beta-tubulin immunofluorescence using epifluorescence microscopy (Figure 1A–F). As a comparison, we also analysed neuroepithelioma MC-IX cells carrying *MYC* amplification in DMs. In >85% of the analysed interphase cells (>1000 cells/cell line scored), DMs were distributed evenly in the nucleus. The remaining nuclei either exhibited DMs restricted to the nuclear periphery (10–14%) or a preferential localisation of DMs to nuclear membrane protrusions or micronuclei (<1%). In contrast, prometaphase and metaphase cells (>100 cells/cell line scored) invariably showed DMs only at the periphery of the prometaphase rosette/metaphase plate. At anaphase, the DMs in all three cell lines remained at the periphery of the chromosome poles and were typically (>90% of DMs) located at, or adjacent to, the termini of the chromosomes (>50 cells/cell line scored). This configuration was also observed in telophase cells. DMs that had detached from the chromosomes were not included in the analyses; these consisted of <10% of observed DMs.

In order to characterize the mitotic distribution of DMs during the cell cycle in three dimensions, we then performed confocal microscopy of mitotic and interphase cells in the three cell lines (>20 mitotic cells and >10 interphase cells scored in each line). DMs were classified as peripherally located if the corresponding FISH signals overlapped with the chromatin border as assessed by DRAQ5 counterstaining (Figs. 1G–K). From prometaphase through telophase, 100% of DMs were located peripherally, close to the chromosome termini. In contrast, <20% of DMs in interphase cells located peripherally, while the remaining DMs were distributed at locations not overlapping with the outer chromatin border. This fluctuation in distribution between interphase and mitosis was observed in all the cell lines, without any significant difference between them in the fraction peripheral DMs at interphase or mitosis.

The mitotic location of DMs adjacent to chromosome termini prompted us to investigate whether DMs co-localized with telomeric repeat sequences in LA-N-5 and CHP-212. Co-hybridization with *MYCN* and TTAGGG-sequence probes assessed by epifluorescence microscopy showed that approximately 80% of *MYCN* signals overlapped with signals from telomeric repeats in mitotic cells from prometaphase through telophase (Figure 1L–N). In contrast, <10% of *MYCN* signals in interphase nuclei typically overlapped with TTAGGG repeat signals. As a comparison, co-hybridization with *MYCN* and pan-centromeric (alpha-satellite sequence) probes was also performed in LA-N-5 and CHP-212. On average, only 4% and 6% of *MYCN* signals, respectively, overlapped with centromere signals in mitotic cells and interphase cells (range 0–13% and 0–16%, respectively; 60 cells analyzed per cell line).

**Figure 2:**
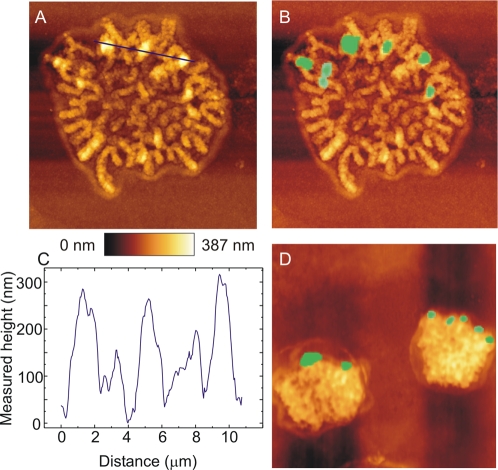
Atomic force microscopy. A. Atomic force microscopy of a CHP-212 prometaphase cell. B. Superimposition of MYCN fluorescence in situ hybridisation (FISH) signals allows localisation of DMs in the AFM image. C. Surface plot of the segment indicated by a blue line in A shows that DMs correspond to surface peaks of approximately 200 nm. D. Superimposed AFM and FISH images show peripheral location of DMs in a late telophase cell. Surface height is shown by the heat map and surface dimensions are indicated by scale bars corresponding to 2 µm in A, B and D.

To validate our fluorescence microscopy data by an additional method, we performed further analysis of DM topography in mitotic CHP-212 cells by combined atomic force microscopy (AFM) and FISH. AFM is a well established technique in material sciences, making it possible to map surface topography with nanometre resolution. Superimposition of FISH images and AFM topography plots made it possible to establish the location of single and clustered DMs with high precision in three dimensions and confirmed the peripheral localisation of DMs at mitosis (Figure 2). Approximately half of the totally 50 DMs analysed were located on the surface of chromosomes, corresponding to peaks of approximately 200 nm in the AFM surface plots. The other DMs were located in grooves between chromosomes. In interphase cells, there was no clear correlation between DMs and surface features by AFM (data not shown). Taken together, our FISH and AFM data thus indicated that DMs underwent repeated topographical fluctuations during the cell cycle, moving rapidly from locations in the nuclear interior at interphase to positions adjacent to the telomeres at prometaphase, where they remained through telophase to finally drift back into the interior of the newly formed interphase nucleus.

### Statistical distribution of DMs at mitosis

**Figure 3:**
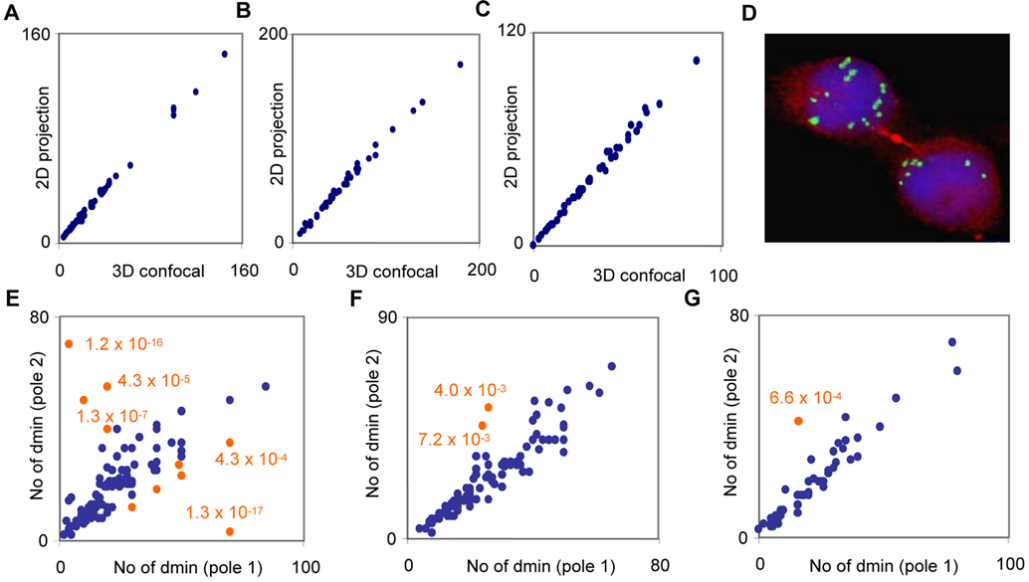
Statistics of DM segregation. A–C. DM copy-numbers in interphase nuclei obtained by scoring 1 µm confocal optical sections (three-dimensional, 3D) plotted against the copy-number obtained by scoring a projected (two-dimensional, 2D) image in LA-N-5 (A), CHP-212 (B) and MC-IX (C). D. Segregation of 7 and 10 DMs, respectively, to each daughter cell at telophase, shown by IF for beta tubulin (red) and FISH for *MYCN* (green). E–G. DM copy-numbers in ana/telophase poles typically follow a binomial distribution (blue data points) in LA-N-5 (E), CHP-212 (F), and MC-IX (G); ana-telophase cells significantly (p<0.01) deviating from the binomial distribution are denoted by orange data points.

We then attempted to assess the statistical distribution of DMs between daughter cells by scoring the DM copy-numbers in opposite ana-telophase poles. To evaluate whether scoring by two-dimensional microscopic analysis was a sufficiently precise method for this, we first scored DMs, detected by *MYCN* or *MYC* probes, in 30 interphase cells from each of CHP-212, LA-N-5, and MC-IX, both by serial confocal sections (3D; 1 AU pinhole) and a single wide confocal section (2D; >5 AU pinhole). The average difference in DM copy number per cell by 2D analysis compared to 3D analysis was very low in each cell line up to a copy-number of approximately 100 (3.4–3.9% of the total DM copy-number; Figure 3A–C). Based on this, we then scored DM copy-number by epifluorescence microscopy in ana- and telophase cells, identified by cross-labelling with beta-tubulin (Figure 3D; 100 cells scored in CHP-212, 100 in LA-N-5, and 50 in MC-IX). Plotting of copy-number data revealed a similar pattern in the three cell lines. An identical DM copy-number in two opposite daughter poles was very rare, but the interpolar differences in copy number were typically small and there was a positive correlation in copy-number between opposite poles (r = 0.53 in LA-N-5, r = 0.92 in CHP-212, r = 0.94 in MC-IX; p<0.05 for all). Our data could readily be approximated by assuming binomial distribution of DMs at mitosis, with a probability of 50% for an individual DM to segregate into each daughter cell (Figure 3E–F). In CHP-212 and MC-IX only 2% of cell divisions deviated significantly (P<0.01) from such a binomial distribution, whereas in LA-N-5, 10% deviated from this distribution.

To assess whether the rare mitoses with a non-binomial DM segregation pattern were reflected by corresponding copy-number heterogeneity in growing cell populations, single cells were then isolated from each cell line and expanded to monoclonal colonies, in which DM copy-numbers were scored in individual cells by interphase-FISH. In total, 20 colonies from each cell line were analysed. With few exceptions, the colonies showed modal DM copy-numbers similar to those of the original cultures. In order not to overestimate the number of cells with copy-numbers produced by a non-binomial distribution, cells that deviated in >20 DM copies from each clone's modal value (outlier cells) were tested against a hypothetical binomial segregation, using the cell with the DM copy-number closest to its own as its presumed post-mitotic sister cell. It has been shown that DMs separate into single minutes during G1-phase [15] and it could therefore not be excluded that outlier cells with higher copy-numbers than a specific colony's modal value represented cells where the DMs had just separated into single-minutes, leading to a duplication of the actual DM copy-numbers. Therefore, only outlier cells with copy-numbers below each colony's modal copy-number were tested. Using this approach, cells with copy-numbers not consistent with binomial segregation were found in 2–6 monoclonal colonies from each cell line (Supplementary Figure S1). Using the total number of cells in each colony to calculate the minimum number of cell divisions that occurred during colony-formation, the frequency of non-binomial DM segregations were approximately 3% (3/88) in CHP-212, 2% (2/100) in MC-IX, and 8% (6/73) in LA-N-5. These values were consistent with the data from direct analysis of ana- and telophase cells, also showing a higher frequency of non-binomial segregation in LA-N-5 (10%) compared to CHP-212 (2%) and MC-IX (2%). Hence, even though non-binomial distributions were rare in the three cell lines, they were sufficiently frequent to generate cells with a DM copy-number strongly deviating from the modal value of the cell population.

### Non-binomial distribution occurs through clustering at high DM copy numbers

**Figure 4:**
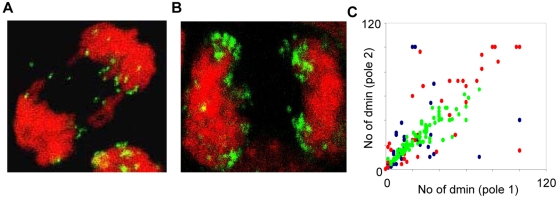
Clustering of DM at high copy-number. A–B. Clustering of DMs at cell division is less frequent in cells with lower (A) than with higher (B) DM copy-number, here exemplified by two anaphase cells. C. DM copy-numbers in CHP-212 ana/telophase poles prior to irradiation (green data points), after irradiation with 2 Gy (red data points), and after irradiation with 4 Gy (blue data points).

One potential explanation of non-binomial DM segregation is detachment of multiple DMs at anaphase, leading to a decreased number of DMs in one of the daughter cells. Further image analysis of the ana- and telophase cells with non-binomial segregation showed that these cells rarely exhibited DMs that detached from the chromosomes, and no more than 4 DMs were observed to be detached in any single cell. This argued against loss of DMs through micronucleus formation as a mechanism for non-binomial segregation. However, the ana- and telophase cells with a non-binomial DM distribution had a higher median DM copy-number than those with a binomial distribution (median 74 vs. 40; P<0.0001 Mann-Whitney test; two-sided; pooled data from the three cell lines). Based on this, we selected 10 ana-telophases with a DM copy-number >50 from each cell line for a detailed analysis by confocal microscopy. This showed that all of the totally 30 analyzed ana-telophases exhibited at least one cluster of DMs at or adjacent to the chromosome termini (Figure 4A and B). The precise number of DMs in each cluster could not be determined at the obtained resolution level, but each cluster appeared to contain at least 5 DMs. In contrast, only approximately one third of ana-telophase cells with 50 or fewer DMs contained clusters, typically containing only two or three DMs.

Previous studies have shown that gamma radiation exposure induces clustering of DMs as well as elimination of DMs through micronucleation [16]. To evaluate whether non-binomial DM distribution could be promoted by radiation-induced clustering of DMs, we subjected CHP-212 cells to ionising radiation (2 Gy and 4 Gy) prior to *MYCN* FISH detection in ana-and telophase cells. Analysis of the DMs that remained attached to chromosomes, often arranged in clusters, indeed showed an elevated frequency of segregations deviating (P<0.01) from the binomial distribution compared to unexposed cells, 2% after 0 Gy, 22% after 2 Gy and 24% after 4 Gy (Figure 4C). Although copy-number estimation could be expected to be more error-prone in these mitoses, with many clustered DMs, our collected data thus indicated that clustering of DMs could be one cause of deviations from the binomial distribution at high DM copy-numbers.

### DM frequency distributions can be modelled by selection imposed on the binomial distribution

**Figure 5:**
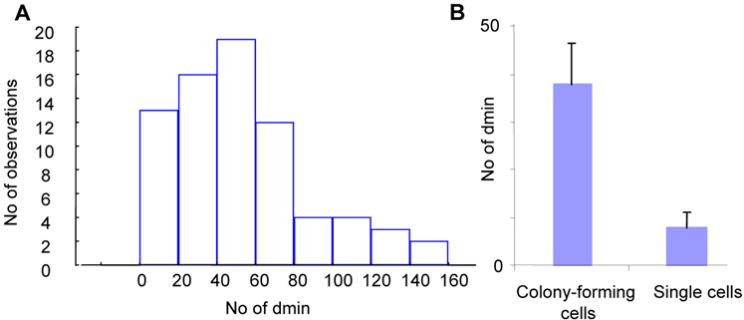
DM copy-number distributions in bulk populations. A. The frequency distribution of DM copy-numbers in near-diploid metaphase cells in LA-N-5 is skewed and differs from a normal distribution. B. Median DM-copy numbers in colony-forming compared to non-colony forming CHP-212 cells; error bars denote 25^th^ and 75^th^ percentiles.

Early studies of DMs in tumour bulk populations by chromosome banding have indicated that their overall copy-number distribution conforms neither to a binomial nor to a Poisson distribution [14]. Assessment of the DM copy-number distribution in metaphase spreads by FISH in CHP-212, LA-N-5, MC-IX and in primary cultures from two fresh neuroblastoma samples revealed a skewed non-binomial (P<0.01; Chi Square test) distribution in all samples, with a tail towards higher copy-numbers (Figure 5A and Supplementary Figure S2). These findings were similar to the distribution previously described in the SEWA-model [14], suggesting a uniform principle behind DM frequency distribution in different cell systems.

**Figure 6:**
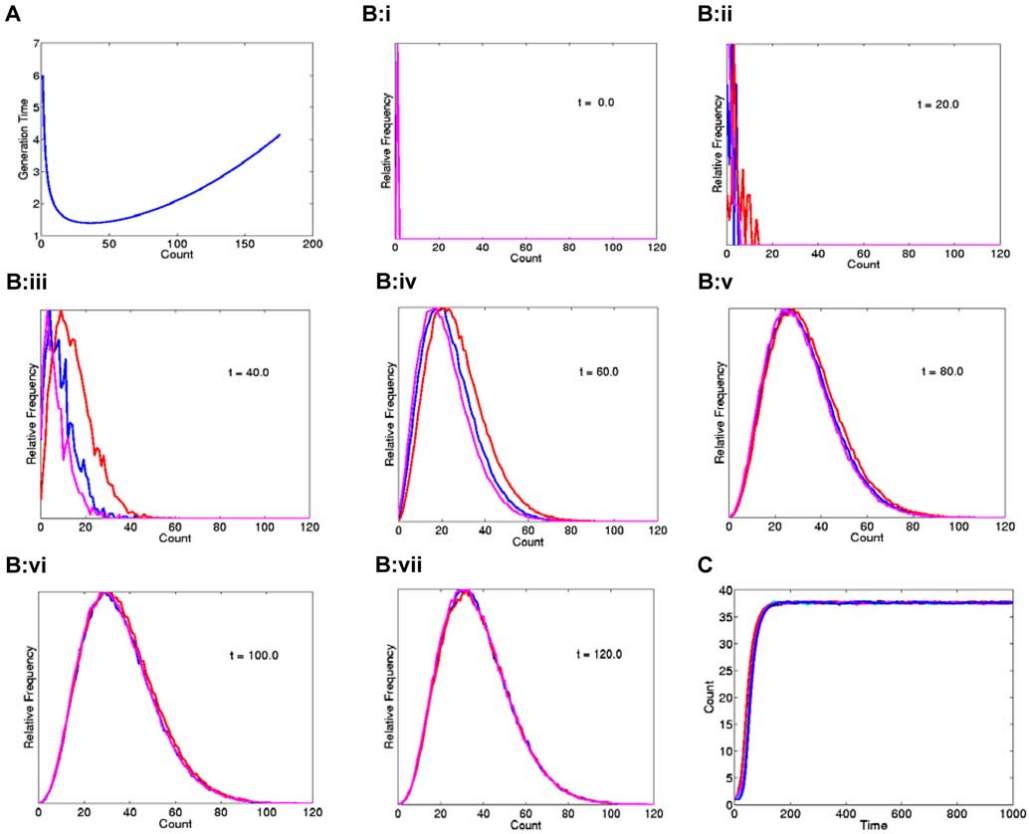
Mathematical modelling. A. Cellular generation time described as a continuous function of the DM-copy number count, with longer generation times for cells with low as well as extremely high DM copy-numbers. Each simulation was initiated with a single cell starting with a DM count equal to 1 at time zero. Cells with 36 DMs have the shortest generation time in the present model, while cells with fewer or extremely high DM-copy numbers proliferate more slowly. B. Convergence towards a skewed distribution for three modelled cell populations (purple, red, and blue curves). C. The equilibrium DM modal numbers for all populations correspond to the copy-number resulting in the shortest generation time in A (n = 36).

It has been well established that a high *MYCN*-copy number corresponds to elevated levels of *MYCN* expression [17]–[19], which in turn results in increased cellular proliferation [2], [20]–[22]. Accordingly, when a colony formation assay was performed for CHP-212, the *MYCN* copy-number by interphase FISH was found to be significantly higher in colony-forming than in single cells (Figure 5B; >20 colonies and >100 single cells evaluated). Thus, proliferative advantages existed, at least *in vitro*, for cells with a high number of DMs, compared to those with a low number. On the other hand, cells with very high (>200 DMs) were extremely rare in our model systems, indicating some degree of selection against cells with an extremely high DM copy-number. Based on this, we hypothesized that cells with a higher DM copy-number would divide more frequently up to a certain copy-number level, where the selection pressure would continuously shift to become less favourable (Figure 6A). A simple algorithm applying these criteria on a model in which DMs segregated in a binomial fashion at mitosis reproduced the skewed frequency distribution observed in our cell lines and tumour biopsies when clonal growth from a single cell containing one DM was simulated (Figure 6B–C). Thus, a very simple model of Darwinian selection superimposed on binomial distribution of DMs at cell division was capable of explaining the frequency distribution of DMs, including a minority cell population with high *MYCN* copy numbers.

### DMs are not irreversibly bound to positionally stable chromosome elements

**Figure 7:**
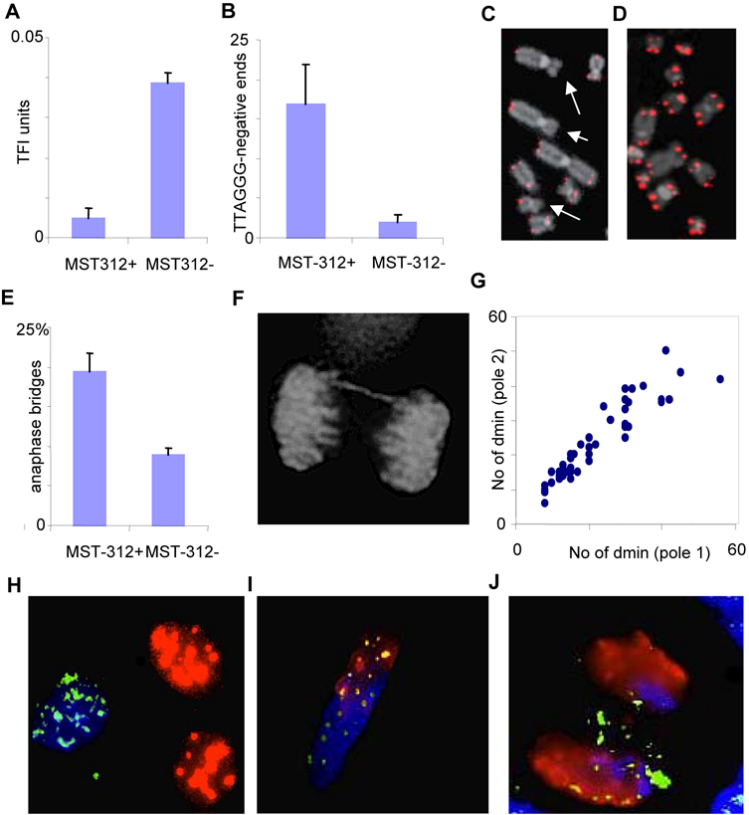
DMs do not bind to telomeres or other specific positional elements. A–B. Mean telomere length (A) assessed by arbitrary telomere fluorescence intensity (TFI) units and dysfunctional telomeres identified as the mean number of TTAGGG-negative chromosome termini (B) in CHP-212 cells exposed (MST312+) and not exposed (MST312-) to a telomerase inhibitor; errors bars denote standard deviations. C–D. A higher number of TTAGGG-negative chromosome termini (arrows) are observed by FISH after telomerase inhibition (C), compared to CHP-212 cells not exposed to the MST-312 inhibitor (D). E–F. Elevated frequency of anaphase bridging after MST-312 treatment. G. The DM distribution between telophase poles does not deviate from a binomial distribution after telomere dysfunction was induced by telomerase inhibition (P>0.01 for all data points). H–J. Co-hybridisation with mouse Cot-1 DNA (red), human Cot-1 DNA (blue) and human *MYCN* (green) probes readily distinguishes between the nuclei of co-cultured murine 3T3 and human CHP-212 cells (H); this labelling protocol also differentiates between murine and human chromatin domains in a murine/human hybrid nucleus (I) and in an anaphase cell (J) where some DMs have migrated into the murine chromatin domains (green-yellow in I and J).

Since DMs were localized very close to telomeres during mitosis, we investigated to what extent the mitotic segregation of DMs was dependent on telomere stability. Neuroblastoma cell lines invariably express telomerase [23], which is believed to be crucial for stabilizing telomere length in transformed cells [24], [25]. To shorten telomeres to a critical length and disrupt telosome function, we treated CHP-212 cells for 14 days with the telomerase inhibitor MST-312 [26], [27]. MST-312 has been shown to inhibit telomerase activity by 47–66% in our culture system [28]. As expected, MST-312 treatment resulted in a significant shortening of telomeres as determined by quantitative FISH with peptide nucleic acid probes against TTAGGG-repeats (Figure 7A). Increased frequencies of chromosome termini without detectable TTAGGG-sequences were also observed, corresponding to critically short, potentially unstable telomeres (Figure 7B–D). Accordingly, MST-312 treated cells showed an elevated frequency of anaphase bridges compared to untreated CHP-212 cells (Figure 7E–F), indicating that telomere shortening induced global chromosomal instability in this cell line. However, when *MYCN*-FISH was performed on MST-312 treated ana-telophase cells, there was no difference in the number of cells deviating from a binomial distribution of DMs (Figure 7G), nor was there an increase in the frequency of DMs that had detached from the chromosomes (one detached DM each in 2/50 cells after MST-312 treatment). Thus, DM segregation did not seem dependent on binding to functional telomeres in human chromosomes.

To further assess whether DMs were bound to any other positionally stable structures on human chromosomes, we used cell fusion by polyethylene glycol to create NB/mouse (CHP-212/3T3) hybrid nuclei. Human- and mouse- specific repetitive DNA sequences and *MYCN*-carrying DMs were then detected by triple colour FISH in these hybrid cells. Hybrid nuclei were obtained in approximately 1∶60 cells. In all the 50 analysed hybrid nuclei, murine and human chromosome domains could be readily distinguished by our FISH strategy (Figure 7H). In these nuclei, 36–76% of DMs were located in murine chromosome domains and there was no difference in the number of DMs located in human domains compared to mouse domains (Figure 7I). In the few mitotic hybrid cells that were observed, DMs attached both to murine and human chromosomes (Figure 7J). Thus, DMs were free to relocate to murine chromosome domains in interspecies hybrid nuclei, indicating that they do not bind irreversibly to a specific positional element on human chromosomes.

## Discussion

*MYCN* amplification is the strongest biological predictor of outcome in NB patients [1]. It has previously been shown that *MYCN* copy-numbers can be highly heterogeneous in NB cell populations. Such genetic heterogeneity may reflect different tumour clones and its role is believed to be under-recognized and underestimated in neuroblastoma biology [29]. In spite of this, the mechanisms behind clonal variability have been little investigated in NB cells. It is known from earlier studies that the DMs typically carrying *MYCN* copies *in vivo* do not separate at anaphase, but remain as double circles until G1-phase [14], [15], [30]. Evidently, a random distribution of DMs at the metaphase-anaphase transition would readily explain clonal variability [18], but whether the events governing DM distribution to daughter cells are really random had not previously been tested experimentally. Furthermore, the randomness function best approximating the behaviour of DMs had not been determined.

In the present study, we show that DMs in NB cells exhibit a regular topographic fluctuation during the cell cycle, translocating rapidly from the nuclear interior to the periphery of the prometaphase rosette at the initiation of cell division, attaching close to the telomeric repeat sequences. For the vast majority of DMs, this attachment was retained from prometaphase through telophase. After reconstitution of the interphase chromatin structure, the DMs relocated to the nuclear interior, possibly to occupy positions at the periphery of chromosome territories [18], separate to single minutes during G1-phase [15], and replicate in S-phase [30] as described. Our findings are highly consistent with previous descriptions of DM behaviour in non-NB model systems, including the human COLO322 [12], [31] and the murine SEWA cell lines [14]. Thus our description of the mitotic topography of DMs mainly serves to confirm that previously suggested models of DM behaviour at cell division are applicable to NB. In contrast, our findings regarding the statistical distribution of DMs are novel and confirm a long held suspicion of the origin of oncogene copy-number heterogeneity.

We showed that the skewed frequency distributions of DM copy-number could be modelled by combining binomial mitotic segregation with a selection pressure favouring cells with a higher DM copy-number up to a certain level. The binomial distribution is the discrete probability distribution of the numbers in a sequence of *n* independent events, each of which yields one out of two possible outcomes with the probability *p*. In the context of DM segregation, *n* corresponds to the DM copy-number at metaphase. Because we assumed that each DM was equally likely to segregate to one of the daughter cells as to the other, *p* was set to 0.5. Our finding that DM copy-numbers can be described by a simple binomial distribution has at least three implications for the evolution of *MYCN* amplicons in NB cells: (1) *MYCN*-copies in DMs segregate independently of each other in a random fashion up to a certain copy-number level. (2) When this level has been reached, the random binomial model is no longer applicable; our experimental data indicate that this level is determined by the copy-number at which DMs start to interact with each other to form clusters, presumably because of the spatial restrictions of the mitotic figures. (3) The modal DM copy-number in the tumour cell population reflects the number of *MYCN*-copies conferring the greatest proliferative advantage under these cells' specific growth conditions. In the established cell lines and tumours used in the present study, this number varied between 25 and 50 DMs.

We cannot completely exclude that non-binomial segregation of clustered DMs also has a role in generating the skewed frequency distribution found in tumour bulk populations. However, non-binomial segregations were relatively rare in our material. Furthermore, they would be more likely to generate discrete sub-populations at non-modal copy-numbers rather than a continuous, skewed distribution. Our findings do not unequivocally explain the negative selection pressure at higher DM copy-numbers, although it points to clustering as a cause of non-binomial segregation at high copy-numbers. Such mitotic clustering should cause dramatic shifts in *MYCN*-copy number after cell division, potentially leading to dysregulation of *MYCN*-dependent intracellular signalling systems and apoptosis [32]. Nevertheless, our mathematical model is supported by several studies showing that *MYCN* amplification confers a proliferative advantage [5], [33]–[35], and also by our own finding of a higher DM copy number among colony-forming CHP-212 cells compared to non-colony forming cells.

Our findings do not explain the molecular mechanisms underlying the topographical fluctuation of DMs during the cell cycle. The co-localisation of DMs and telomeric TTAGGG-repeats at mitosis could suggest that DMs bind to parts of the telosome multiprotein complex. However, disruption of telomere stability by telomere shortening induced by MST-312 failed to perturb the binomial segregation of DMs at cell division. Even though our studies do not fully exclude that DMs bound to the telomeres that remained intact after telomerase inhibition, our data speak against a specific interaction between DMs and the telosome complex. This was further supported by the finding that not all DMs co-localised with telomere repeats at cell division and also by our confocal microscopy and AFM images showing that several DMs were localised to interstitial segments close to the termini, rather than to the termini *per se*. A previous study has demonstrated that DMs are typically located at the periphery of folded chromosome territories in interphase nuclei [18]. It is possible that the centripetal force acting on centromeres during formation of the prometaphase rosette results in a centrifugal movement of DMs relative to the chromosomes as DMs do not interact with the mitotic spindle. The peripheral translocation of DMs at prometaphase formation would then be caused by DMs sliding longitudinally along the chromosomes as the interphase chromosome territories condense at prometaphase and are pulled centrally by the spindle fibres. Our findings are consistent with those of a previous study, based on the COLO322 cell line, showing that DMs were repelled from the spindle poles while they attached to the chromosome periphery [11]. Disrupting microtubule organization eliminated such peripheral localization of DMs in this model system, but it did not affect their association with chromosomes, indicating that a microtubule-mediated antipolar force would be responsible for the mitotic fluctuation of DMs. Together with our data, these findings imply that unspecific, weak biophysical forces cause the attachment of DMs to chromosomes during mitosis. This is supported by our finding that DMs could move freely in the interphase nuclei of interspecies hybrids.

The present study is one of a few showing a specific mechanism causing genetic heterogeneity by random events, which in turn can form the substrate for selection according to Darwinian principles, ultimately leading up to oncogene amplification and an increased proliferation of tumour cells. Furthermore, by clarifying the statistical principles of *MYCN* amplicon segregation, we have now acquired a precise standard against which the effects of potential anti-neuroblastoma drugs acting through *MYCN* amplicon elimination can be compared. The mechanisms by which DMs bind to chromosomes in NB clearly need to be studied further, particularly as the molecules participating in this attachment would be attractive therapeutic targets. If the binding of DMs to chromosomes could be efficiently, and specifically, inhibited, this could be an efficient way of eliminating *MYCN* amplicons from NB cells, thus causing growth reduction and apoptosis [5]–[7]. The present study may provide a first step in identifying target proteins for such drugs, implicating weak biophysical forces rather then covalent attachments and binding to broadly distributed chromosome bio-molecules rather than specific binding to the telosome nucleoprotein complex.

## Materials and Methods

### Cell lines and cell culture

The study was reviewed and approved by the Ethics Review Board at Lund University Hospital, Sweden. The NB cell-lines CHP-212 and LA-N-5, the neuroepithelioma cell-line MC-IX, and mouse 3T3 cells were obtained from the American Type Culture Collection. Chromosome preparations from NB tumour biopsies and bone marrow infiltrates were obtained from the Department of Clinical Genetics in Lund. Cell culturing, harvest and chromosome preparation for FISH were according to standard methods [36]. All cells were cultured in DMEM∶F12 1∶1 supplemented with 10% foetal bovine serum and antibiotics. Single-cell clones were made from trypsinized single cell suspensions and plated in collagen- and fibronectin-coated chamber slides (5,000–10,000 cells/slide). Attaching cells were cultured for two weeks under standard conditions. The analysis was limited to colonies with a circular growth pattern, growing separate from other colonies, in order to avoid scoring colonies originating from different cells. Cell lines subjected to ionizing radiation were harvested for analysis 5–10 after irradiation, when mitotic figures were first visible by microscopic inspection.

### FISH analysis

DMs were detected by single-copy probes for *MYCN* and *MYC* (Abbott Molecular Inc., Des Plaines, IL) and telomeric repeat sequences by fluorescein-conjugated (CCCTAA)^3^ peptide nucleic acid probes as described [37]. Human and murine DNA sequences were identified by hybridisation with Cy3-labelled mouse Cot-1 and biotin-Cy5-labelled human Cot-1, respectively (Invitrogen, Stockholm, Sweden). The number of DMs was calculated manually in digital images acquired by a CCD camera coupled to an epi-fluorescence microscope. For combined immunofluoroscence and FISH, cells were fixed in −20°C methanol for 5–10 minutes and air-dried. Slides were then rehydrated in PBS containing 1% bovine serum albumin (BSA) for at least 30 min. Beta-tubulin was detected by the monoclonal antibody TUB2.1 (Sigma-Aldrich, St. Louis, MO), conjugated to Cy3, diluted 1∶100 in 1% BSA/PBS and incubated with the target cells for 60 min at room temperature. After incubation, target cells were washed twice in 1% BSA/PBS and antibody conjugation was fixed by immersion in 1% formaldehyde for 5 min. After washing in PBS and dehydration in ethanol, FISH was performed by standard protocols without prior enzymatic pre-treatment.

### Confocal microscopy

Cells were fixed according to Solovei et al. [18]. Confocal images were obtained on a Zeiss LSM 510 META system with an inverted Zeiss Axiovert 100 M microscope and LSM 510 META software version 3.2 (Carl Zeiss, Jena, Germany). FITC was excited with the 488 nm line of a krypton-argon laser and emission was collected through a Plan-Fluar 100x/1.45 NA objective using a band-pass 505–550 nm filter. DRAQ5-labelled chromatin (Biostatus Ltd, Shepshed, UK) was excited by the 633 nm line of a helium-neon laser and emission was collected using a band-pass 644.5–719.4 filter. The pinhole was adjusted to 1 AU (Airy units) when acquiring confocal images and set to 5 AU or above for two-dimensional images.

### Combined AFM and FISH

Combined AFM and optical microscopy investigations were performed using a JPK Nanowizard II AFM with the JPK Life Science stage mounted on an inverted optical microscope (Nikon TE2000-U; JPK Instruments AG, Berlin, Germany). For all measurements, a Nikon CFI Plan Apo VC 60x, 1.40 DIC oil immersion objective was used. For epi-fluorescence a mercury light source was used in combination with standard filter blocks: DAPI (Ex 340-380nm, DM 400nm, BA 435-485nm), TRITC (Ex 540/25nm, DM 505nm, Em 605/55nm). A standard CCD camera was used for the digital imaging acquisition. Prior to AFM imaging, the samples were inspected by phase contrast microscopy to locate the regions of interest as well as for precise location of the AFM tip. The cantilever was placed above the selected region using the positioning screws on the stage before approaching the tip to the sample surface. For AFM imaging, standard noncontact cantilevers were used in intermittent contact mode to reduce the lateral forces during scanning. The scan speeds were typically 0.3–0.5 Hz when acquiring images with 512×512 pixels. AFM and FISH images were superimposed using AdobePhotoshop.

### Interspecies cell hybrids

Cytoplasmic membrane fusion was achieved in sub-confluent cultures containing CHP-212 and 3T3 cells at equal proportions by washing in serum-free medium, followed by treatment with 50% polyethylene glycol (PEG 6000, BDH Chemicals Ltd., Poole, UK) for 2 min. PEG-treated cells and untreated control cultures were then grown in DMEM∶F12 1∶1 supplemented with 10% foetal bovine serum and antibiotics for 3–4 days before harvest.

### Telomerase inhibition

The MST-312 telomerase inhibitor (Calbiochem/EMD Biosciences, Madison, WI) was diluted in DMSO and used at a noncytotoxic concentration of 1.0 µmol/L (25). To maintain a stable concentration of MST-312, the medium was changed every third day. Cultures exposed only to DMSO were cultured in parallel as controls. Colony formation assay was done by plating 10 000 cells on chamber slides. The rate of population doubling was measured by colony formation assay at regular intervals with colonies defined as groups of >10 cells.

### Mathematical modelling

The time between divisions for a cell with n DMs was set to 1+10/(1+n)+nˆ2/10000, where the term 1 was the lower limit of the inter-mitotic time, and the term 10/(1+n) simulated the growth advantage of cells with high DM copy-numbers. 1/(n+1) was used instead of 1/n so that cells with 0 DMs would obtain a finite time to the next division. The term nˆ2/10000 was used to simulate a penalty on cells with very high DM counts. After each mitotic cycle, the n for each cell was doubled and then distributed randomly according to a binomial function between the two daughter cells. To keep computations limited we capped the cell population at 100,000 by random deletion. Each simulation was initiated with a single cell starting with a DM count equal to 1 at time zero. Matlab (MathWorks, Kista, Sweden) programming was used to simulate the growth of each population according to these rules.

## Supporting Information

Figure S1 Single cell colonies. DM interphase copy-number distributions in single-cell-derived colonies from LA-N-5, CHP-212, and MC-IX. Scored interphase nuclei correspond to red and grey circles, respectively, of which only the former were included in the statistical analysis (see text). Variations in DM copy-number that were not explained (P<0.01) by a binomial distribution are marked by arrows.
(0.57 MB TIF)

Figure S2 DM copy-numbers. The DM frequency distribution in near-diploid metaphase cells in the CHP-212 and MC-IX cell lines, and in biopsies from one primary NB (Patient 1), and one bone marrow NB metastasis (Patient 2). Similar to SK-N-5, the distributions are skewed towards higher copy-numbers and differ from a normal distribution (P<0.01; Chi Square Test).
(0.10 MB TIF)
