# Supplementary figures and images for: Binomial Mitotic Segregation of MYCN-Carrying Double Minutes in Neuroblastoma Illustrates the Role of Randomness in Oncogene Amplification
Source: PLoS One. 2008 Aug 29;3(8):e3099. doi: 10.1371/journal.pone.0003099 (PMC2518122; doi:10.1371/journal.pone.0003099)

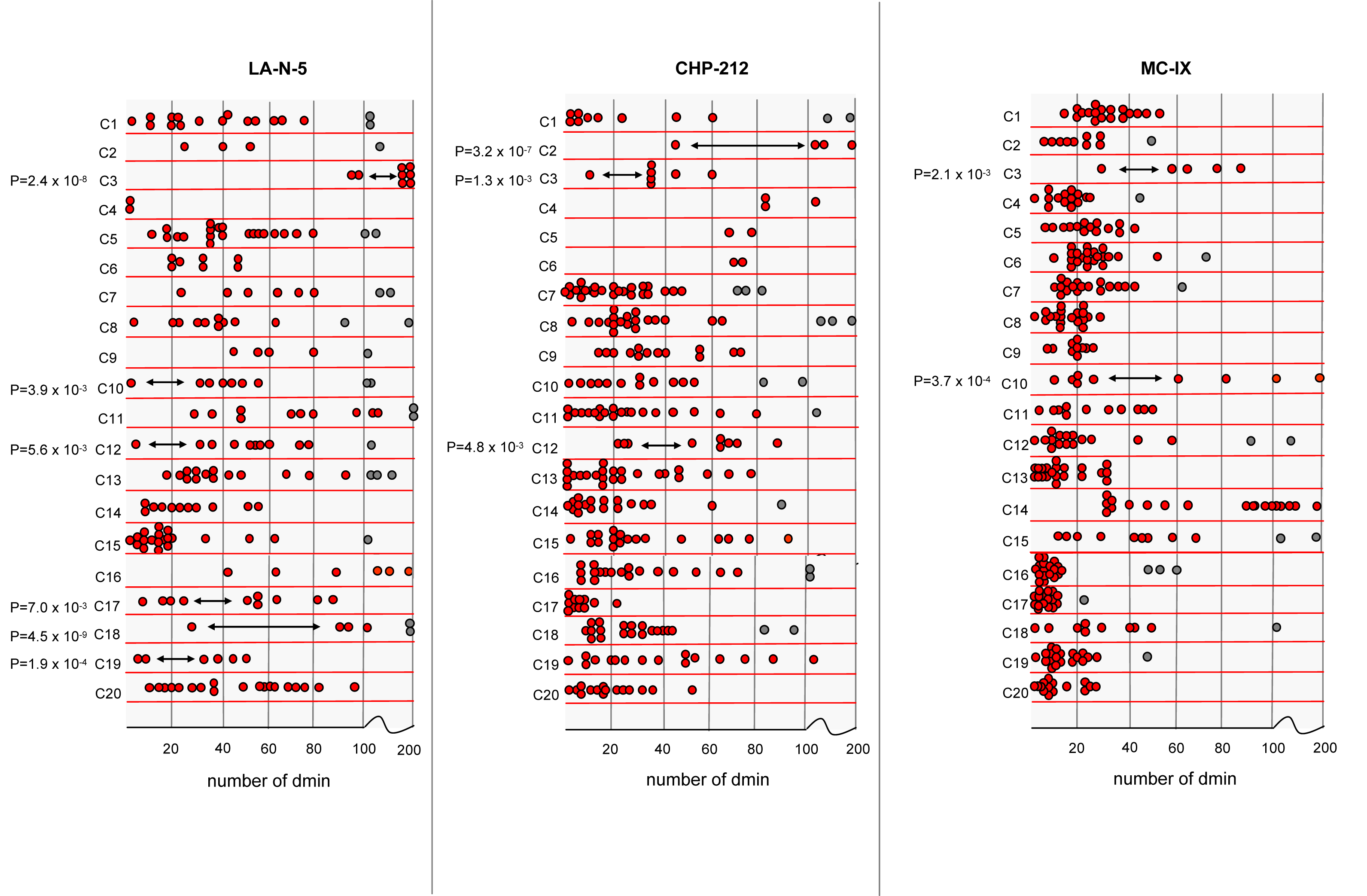

Supplement: Figure S1 — Single cell colonies. DM interphase copy-number distributions in single-cell-derived colonies from LA-N-5, CHP-212, and MC-IX. Scored interphase nuclei correspond to red and grey circles, respectively, of which only the former were included in the statistical analysis (see text). Variations in DM copy-number that were not explained (P<0.01) by a binomial distribution are marked by arrows. (0.57 MB TIF) [file pone.0003099.s001.tif]

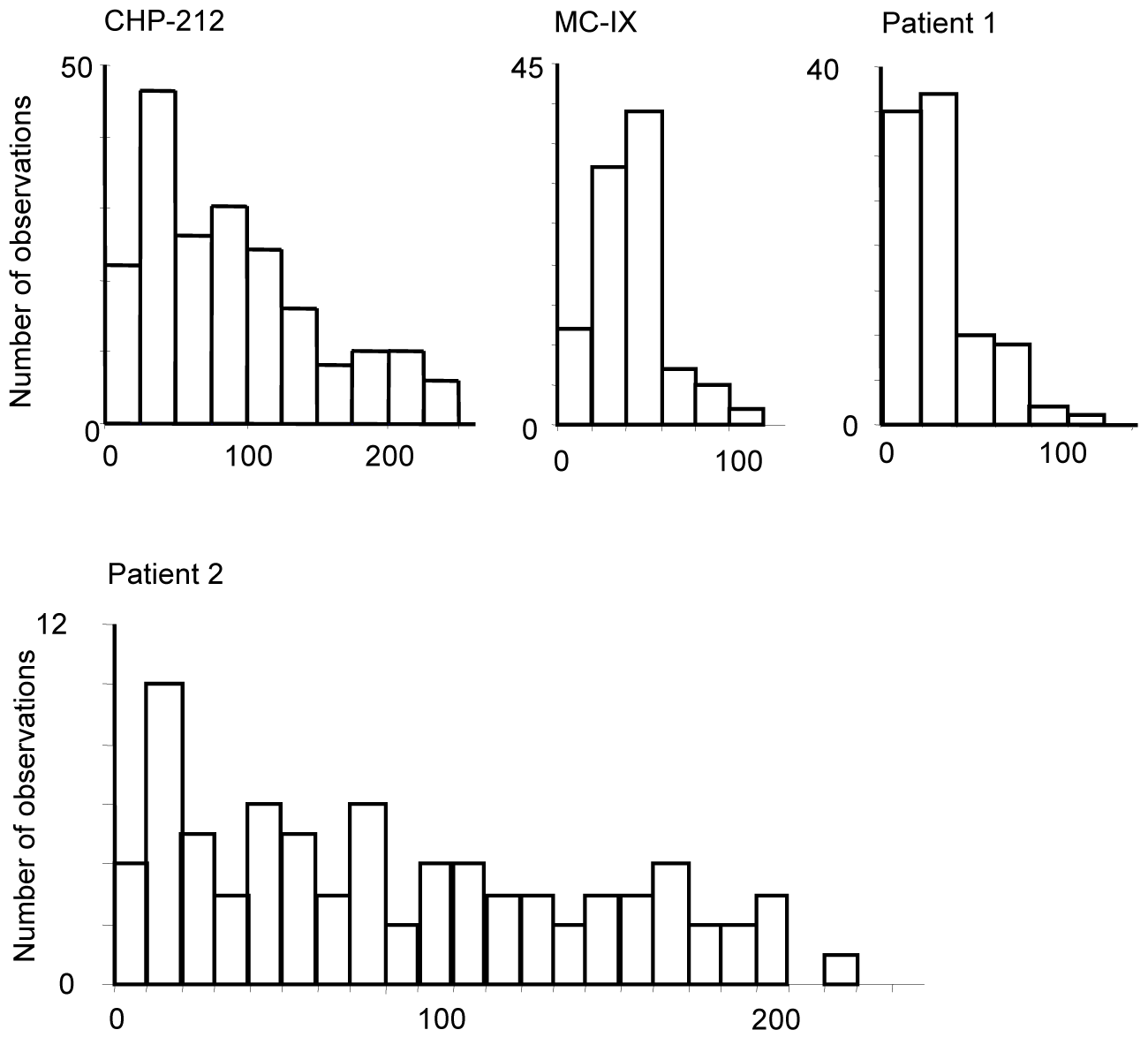

Supplement: Figure S2 — DM copy-numbers. The DM frequency distribution in near-diploid metaphase cells in the CHP-212 and MC-IX cell lines, and in biopsies from one primary NB (Patient 1), and one bone marrow NB metastasis (Patient 2). Similar to SK-N-5, the distributions are skewed towards higher copy-numbers and differ from a normal distribution (P<0.01; Chi Square Test). (0.10 MB TIF) [file pone.0003099.s002.tif]
